# Allelopathic Effects of Extracts from Different Sorghum Parts on Giant Foxtail

**DOI:** 10.3390/plants15172628

**Published:** 2026-08-28

**Authors:** Mengyao Liu, Haonan Wang, Haoyan Shen, Jiaxin Xie, Peiyao Li, Xi’e Song, Yinyuan Wen, Chunyan Hu, Yongqing Ma, Shuqi Dong

**Affiliations:** 1 College of Agriculture, Shanxi Agricultural University, Taigu 030800, China; lmy991215@163.com (M.L.); wanghaonan2004@163.com (H.W.); shy04091@163.com (H.S.); xjx13525275169@163.com (J.X.); peiyaoli666@163.com (P.L.); sxndsxe@163.com (X.S.); wenyinyuan@126.com (Y.W.); 2 College of Plant Protection, Shanxi Agricultural University, Taigu 030800, China; 3 College of Soil and Water Conservation Science and Engineering (Institute of Soil and Water Conservation), Northwest A&F University, Yangling 712100, China; mayongqing@ms.iswc.ac.cn

**Keywords:** sorghum, giant foxtail, water extract, allelopathy, agronomic traits, antioxidant enzyme activities

## Abstract

Giant foxtail is a common weed in foxtail millet fields, and continuous cropping of foxtail millet increases its occurrence. Rotating foxtail millet with sorghum is a major practice in the dry farming regions of northern China. Given this situation, this study combined Petri dish germination tests with pot experiments to clarify the allelopathic effects of water extracts from different sorghum parts on giant foxtail. Distilled water (SCK) was used as the control, and treatments included stock solution (S1), as well as 10× dilution (S2), 50× dilution (S3), and 100× dilution (S4) of water extracts prepared from sorghum roots, stems, leaves, and rhizosphere soil. The results showed that water extracts from all sorghum parts exhibited promotion of giant foxtail seed germination at higher dilutions and inhibition at lower dilutions. The S3 treatment of rhizosphere soil extract achieved a germination rate of 62.22%, which was significantly higher than that of the SCK by 40.00%. The germination energy of root extract S1 was only 4.00%, which was significantly lower than that of the SCK by 86.67%. The S3 treatment showed the strongest allelopathic promotion effect. All dilution treatments promoted the plant height, stem diameter, and above ground fresh and dry weight of giant Foxtail, and these effects increased initially and then stabilized over time. The root extract exhibited higher allelopathic activity than the other extracts. The activities of superoxide dismutase (SOD), peroxidase (POD), and catalase (CAT) first increased and then decreased as the dilution factor increased, while the S1 treatment inhibited enzyme activities and caused an imbalance in the antioxidant system. Sorghum root water extract had a significant effect on CAT activity in giant foxtail, with an increase of 98.82% under the S3 treatment compared with the SCK at 40 d. Malondialdehyde (MDA) content increased with decreasing dilution factor, and the undiluted extract induced membrane lipid peroxidation damage. In conclusion, the allelopathic effects of sorghum extracts on giant foxtail were closely related to the dilution level. Higher dilutions activated the antioxidant defense system and promoted growth, while lower dilutions disrupted enzyme system balance and exacerbated membrane damage. These findings provide basic data and theoretical support for the development and utilization of sorghum allelochemicals and for the green control of giant foxtail.

## 1. Introduction

Foxtail millet (*Setaria italica* (L.) Beauv) is an annual crop belonging to the genus Setaria in the grass family. It is one of the oldest cultivated crops in the world [1,2]. China is the center of origin for foxtail millet, with a domestication history of approximately 8000 years. Today, China remains the world’s largest producer and consumer of foxtail millet, accounting for 80% of the global cultivation area and 90% of its total production. The crop is grown on approximately 1.4 million hectares annually in China, with major producing regions concentrated in Hebei, Shanxi, eastern Inner Mongolia, and the northeastern provinces. The potential planting area for foxtail millet is estimated at 2.38 million hectares, capable of producing 9.92 million tons [3]. It shows strong tolerance to drought, heat, poor soil, and saline–alkali conditions [4,5], and it is mainly grown in the arid and semi-arid regions of northern China [6]. However, foxtail millet fields have many weed species, with grasses and broadleaf weeds growing together [7,8]. Giant foxtail (*Setaria faberi* Herrm.) is one of the most harmful weeds in foxtail millet fields. It belongs to the genus Setaria and is considered a variety of green foxtail [9]. As a stubborn and noxious weed, giant foxtail causes serious problems. It competes with the crop for water, nutrients, light, and space, which leads to yield loss [10]. It can also carry pests and diseases, which reduce crop quality. The competition between giant foxtail and foxtail millet is not limited to physical resources like light, water, and nutrients. It also involves complex chemical ecological interactions [11]. Because these two plants are closely related, giant foxtail has strong tolerance to the allelochemicals released by foxtail millet. This adaptation, developed through long-term co-evolution, makes it more challenging to use allelopathy-based biological control strategies against this weed.

Studies have shown that the allelopathic rice variety PI312777 can significantly suppress the growth of barnyard grass, and its residual effect in the soil can last into the following crop [12]. In wheat and maize rotation systems, grasses and legumes used as previous crops have the strongest suppressive effects on associated weeds. Planting maize or sorghum before wheat can reduce weed biomass in wheat fields and increase crop yield [13]. Sorghum roots release sorgolactone, which accumulates in the soil and shows stronger weed suppression in the second year [14]. For legume crops, a review suggested that in semi-arid tropical regions, reasonable rotation sequences of cereals, legumes, oilseeds, and commercial crops can use allelopathy to control weeds effectively and reduce herbicide use [15]. Although synthetic chemical herbicides currently play a key role in weed control, their long-term use has raised widespread concern about negative effects, including chemical runoff, changes in soil physical and chemical properties, and the development of herbicide-resistant weeds [16,17]. Currently, only seven herbicides are registered for use in foxtail millet fields, and foxtail millet is very sensitive to chemical herbicides. Manual weeding is still the main method of weed control, and requires heavy labor and high costs [18,19]. After continuous cropping of foxtail millet, the occurrence of noxious weeds such as giant foxtail increases significantly, becoming an important factor that limits the improvement of foxtail millet yield and quality [20]. There may be complex chemical ecological interactions between foxtail millet and giant foxtail. Rotating foxtail millet with sorghum (*Sorghum bicolor* (L.) Moench) is a major cropping practice in the dry farming regions of northern China. The roots of these two crops use soil nutrients at different soil layers, which can reduce the buildup of diseases caused by continuous cropping of a single grass species. Through measures such as reasonable rotation, soil improvement, and disease control [21], the problems caused by continuous foxtail millet cropping can be effectively reduced.

Understanding the allelopathic effects of sorghum on giant foxtail and the related physiological and biochemical mechanisms has both theoretical and practical importance for developing ecological weed control techniques based on allelopathy in foxtail millet rotation systems. Identifying the most active sorghum organs and the effective dilutions for suppressing giant foxtail can provide basic data and theoretical support for foxtail millet–sorghum rotation and for green weed control in foxtail millet fields. This work also expands the application of allelopathy in ecological weed management and provides technical support for promoting the green development of the foxtail millet industry.

## 2. Results

### 2.1. Effects of Water Extracts from Different Sorghum Parts on Giant Foxtail Seed Germination

**Figure 1:**
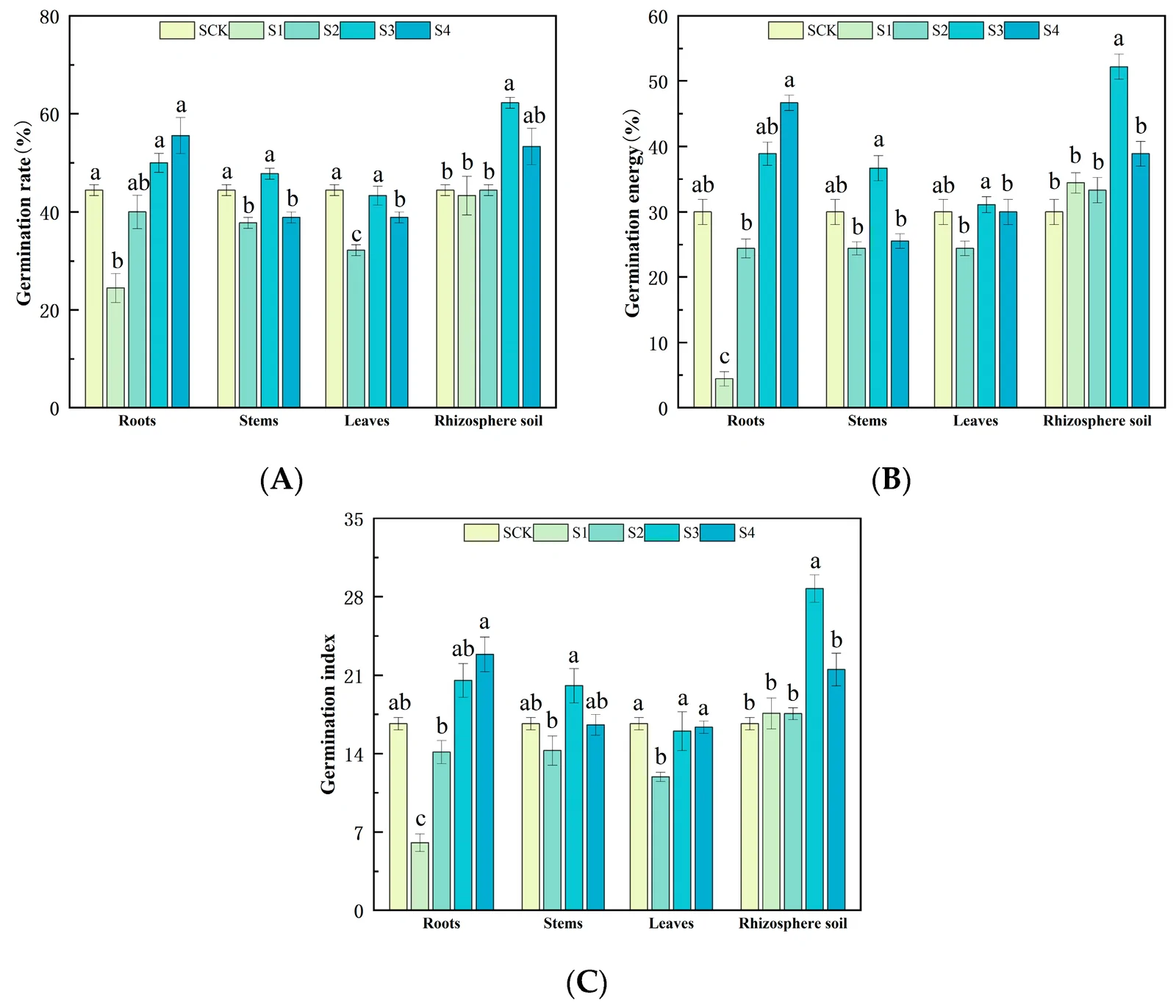
Effects of water extracts from different sorghum parts on germination parameters of giant foxtail. (**A**) Germination rate; (**B**) germination energy; (**C**) germination index. During the germination test, no giant foxtail seeds germinated under the S1 treatment of stem and leaf water extracts, resulting in missing data for these treatments. Comparisons among different dilutions within the same plant part are shown, with lowercase letters indicating significant differences (*p* < 0.05).

The effects of S1 (stock solution) and the diluted extracts from different sorghum parts on giant foxtail seed germination rate differed significantly, generally showing a trend of promotion at higher dilutions and inhibition at lower dilutions. Compared with the SCK, the root S1 (stock solution), stem S2 (10× dilution) and S4 (100× dilution), and leaf S2 and S4 treatments reduced germination rates by 45.00%, 14.99%, 12.49%, 27.50%, and 12.49%, respectively, and all these differences were significant. The S3 (50× dilution) rhizosphere soil extract achieved a germination rate of 62.22%, which was significantly higher than that of the SCK (distilled water) by 40.00%. The other dilutions also generally had lower germination rates than the SCK (Figure 1A).

The germination energy of root extract S1 was only 4.00%, which was significantly lower than that of the SCK by 86.67%, making it the strongest inhibitory treatment. The S4 treatment increased germination energy by 56.67% compared with the SCK. For stem extracts, S3 increased germination energy by 22%, while S2 and S4 decreased it by 18% and 15%, respectively. For leaf extracts, S2 decreased germination energy by 19% compared with the SCK, while the other dilutions showed no significant differences from the SCK. For rhizosphere soil extracts, S1, S2, and S4 showed no significant differences from the SCK, while S3 significantly increased germination energy by 73.33% compared with the SCK (Figure 1B).

Germination index is an important indicator of seed vigor, and its value is positively related to seed vigor. For root extracts, the S1 treatment had a germination index of 6.07, which was significantly lower than that of the SCK by 63.59%, making it the strongest inhibitory treatment. The S3 and S4 treatments increased the germination index by 23.3% and 37.19%, respectively. For stem extracts, S3 increased the germination index by 20% compared with the SCK, while S2 decreased it by 14.4%. Leaf extract S2 significantly decreased the germination index by 28.44%. Rhizosphere soil extract S3 significantly increased the germination index by 72.35% (Figure 1C).

### 2.2. Effects of Water Extracts from Different Sorghum Parts on Root and Shoot Growth of Giant Foxtail After Seed Germination

The root extract S1 had the strongest inhibitory effect, with root length and root fresh weight significantly decreased by 73.39% and 44.12% compared with the SCK, while shoot fresh weight was significantly increased by 45.58%. The other dilutions showed varying degrees of promotion. For stem extracts, S2 and S3 showed no significant differences in root length, root fresh weight, or shoot length compared with the SCK. S4 significantly promoted growth, with root length, root fresh weight, shoot length, and shoot fresh weight increased by 26.06%, 67.65%, 66.67%, and 44.19%, respectively. For leaf extracts, S2 significantly decreased root length by 32.66% compared with the SCK, but root fresh weight and shoot length showed no significant changes, while shoot fresh weight increased by 26.98%. S3 and S4 showed significant promoting effects. For rhizosphere soil extracts, S1 significantly promoted root fresh weight, shoot length, and shoot fresh weight, with increases of 79.41%, 90.67%, and 63.72%, respectively. S2 significantly promoted root length and root fresh weight, with increases of 46.79% and 151.96%. S3 decreased shoot length and shoot fresh weight by 18.67% and 18.60% compared with the SCK. S4 significantly promoted root length and root fresh weight, with increases of 25.69% and 87.25% (Table 1).

### 2.3. Allelopathic Effects of Water Extracts from Different Sorghum Parts on Giant Foxtail Seed Germination

The S1 treatment of root extracts had the strongest inhibitory effect, with an SE value of −0.262. The RI values for germination rate, root length, and root fresh weight were all negative, and the RI for root length reached −0.733, indicating that root extract S1 had the strongest allelopathic inhibitory effect on root length. Only shoot fresh weight was slightly promoted. The SE values of all dilutions were positive, indicating promoting effects. For stem extracts, SE values increased as the dilution factor increased. The S3 treatment had an SE of 0.031, showing weak promotion, while S4 had an SE of 0.238, showing relatively strong promotion. For leaf extracts, SE values first increased and then decreased as the dilution factor increased, changing from negative to positive, which means the effect shifted from inhibition to promotion. The S2 treatment had an SE of −0.050, showing inhibition, with root length being notably inhibited (RI = −0.326). The S3 and S4 treatments had SE values of 0.313 and 0.290, respectively, both showing promotion. All treatments of rhizosphere soil extracts had positive SE values, all showing promoting effects. In summary, the strongest allelopathic inhibitory effect on giant foxtail seed germination was observed in the root extract S1 treatment, with an SE of −0.262. The leaf extract S2 treatment showed weak inhibition, with an SE of −0.050. Both treatments inhibited germination rate and root length (Table 2).

### 2.4. Effects of Water Extracts from Different Sorghum Parts on the Agronomic Traits of Giant Foxtail

**Figure 2:**
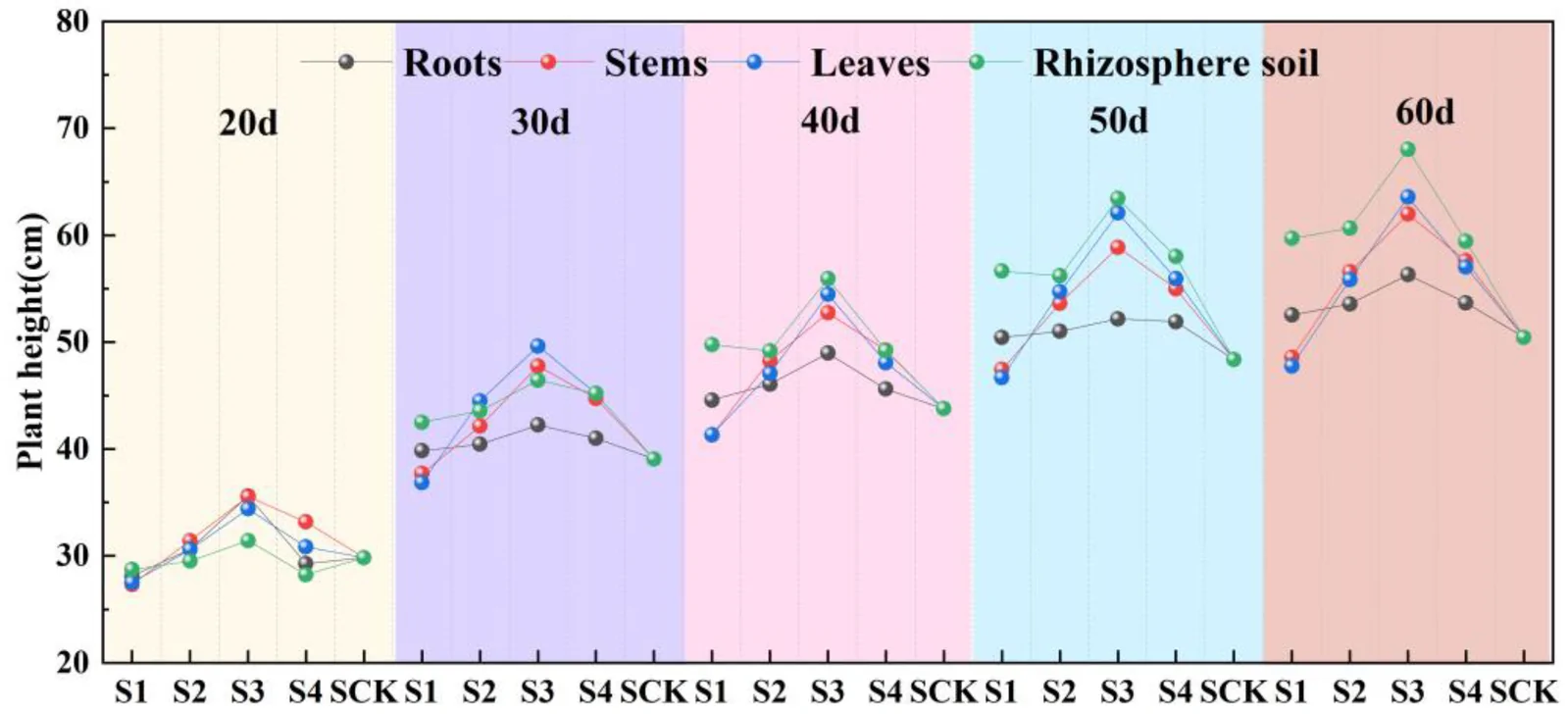
Effects of water extracts from different sorghum parts on the plant height of giant foxtail.

The effect of sorghum water extracts on giant foxtail plant height showed that the S1 treatment inhibited growth, while the diluted extracts promoted growth. As the dilution factor increased, the promoting effect first increased and then decreased. Among the dilutions, the S3 treatment had the strongest promoting effect, followed by S2, while S4 had the weakest effect. Over time, the promoting effect generally increased. Under the S3 treatment, root extract increased plant height by 7.85% to 19.46%, stem extract by 19.10% to 22.86%, leaf extract by 15.31% to 28.33%, and rhizosphere soil extract by 5.36% to 34.91% compared with the SCK. Under the S1 treatment, stem extract decreased plant height by 8.48% at 20 d, and rhizosphere soil extract decreased it by 17.15% and 18.20% at 50 d and 60 d, respectively. The S4 treatment of rhizosphere soil extract significantly decreased plant height by 19.51%, 21.26%, and 17.85% at 40 d, 50 d, and 60 d, respectively (Figure 2).

**Figure 3:**
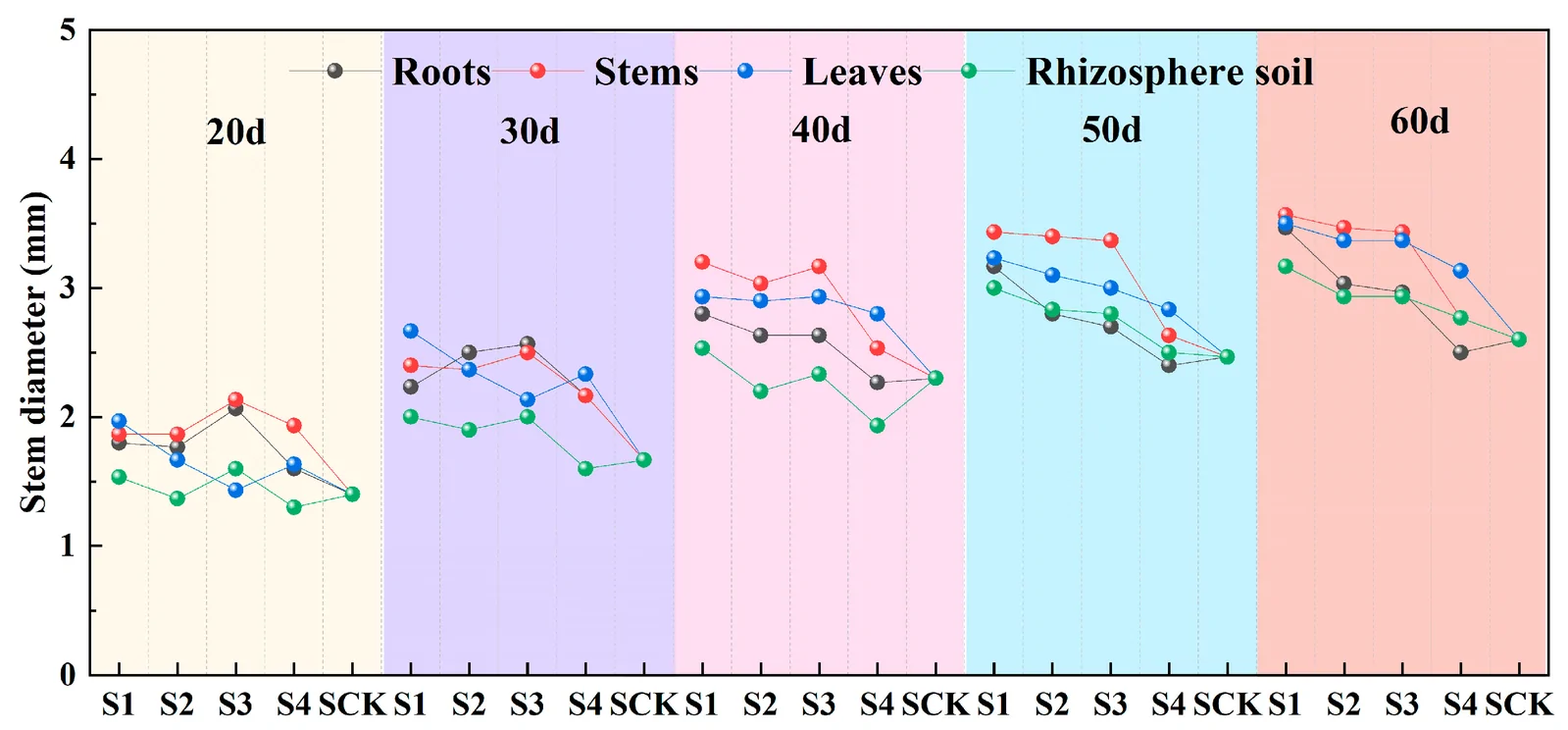
Effects of water extracts from different sorghum parts on the stem diameter of giant foxtail.

The effect of sorghum water extracts on giant foxtail stem diameter showed that all S1 treatments significantly promoted stem diameter. As the dilution factor increased, the promoting effect decreased, and some S4 treatments showed inhibition. Under the S1 treatment, leaf extract increased stem diameter by 60.00% at 30 d compared with the SCK. At 20 d, root, stem, and leaf extracts increased stem diameter by 28.57%, 33.33%, and 40.48%, respectively. The S2 treatments still generally showed significant promotion. The promoting effect of S3 treatments decreased. Root extract increased stem diameter by 47.62% at 20 d, but leaf extract increased it by only 2.38% at 20 d, and this difference was not significant. Under the S4 treatment, rhizosphere soil extract decreased stem diameter by 7.14%, 4.00%, and 5.94% at 20 d, 30 d, and 40 d, respectively, compared with the SCK. Most other S4 treatments showed no significant differences (Figure 3).

All extracts from different sorghum parts promoted the aboveground fresh weight of giant foxtail. The S3 treatment had the best effect and showed the largest increases on multiple occasions. Leaf extract increased aboveground fresh weight by 93.53% at 40 d, and stem extract increased it by 118.61% at 20 d. The S1 treatment of leaf extract showed an exceptionally strong effect at 30 d, increasing aboveground fresh weight by 73.98% compared with the SCK. At most other time points and for other plant parts, the promoting effects of S1 were weaker than those of the diluted treatments. At 20 d and 30 d, extracts from all parts showed relatively strong promoting effects. Although some treatments fluctuated over time, the S3 treatment maintained significant promoting effects across all time points. Overall, stem and leaf extracts had better promoting effects than root extracts (Table 3).

Under the S3 treatment, root extract increased aboveground dry weight by 61.82%, 114.75%, 56.04%, 89.24%, and 67.54% from 20 d to 60 d compared with the SCK. Stem extract increased it by 92.73%, 74.86%, 77.12%, 73.11%, and 55.36%. Leaf extract increased it by 127.27%, 74.32%, 92.29%, 140.44%, and 116.23%. Rhizosphere soil extract increased it by 130.91%, 128.42%, 58.87%, 62.75%, and 48.41%. All these increases were significant. Among them, the S3 treatment of leaf extract showed the most prominent promoting effect at 50 d. The S1 treatments also showed significant promotion. Root extract S1 increased aboveground dry weight by 83.61% at 30 d, and leaf extract S1 increased it by 81.45% at 60 d. The S2 and S4 treatments generally showed promoting trends but with smaller magnitudes. Stem extract S2 decreased aboveground dry weight by 2.19% at 30 d compared with the control, and rhizosphere soil extract S4 decreased it by 3.64% at 20 d. Both decreases were relatively small (Table 4).

### 2.5. Effects of Water Extracts from Different Sorghum Parts on the Activities of SOD, POD, CAT, and MDA Content in Giant Foxtail

**Figure 4:**
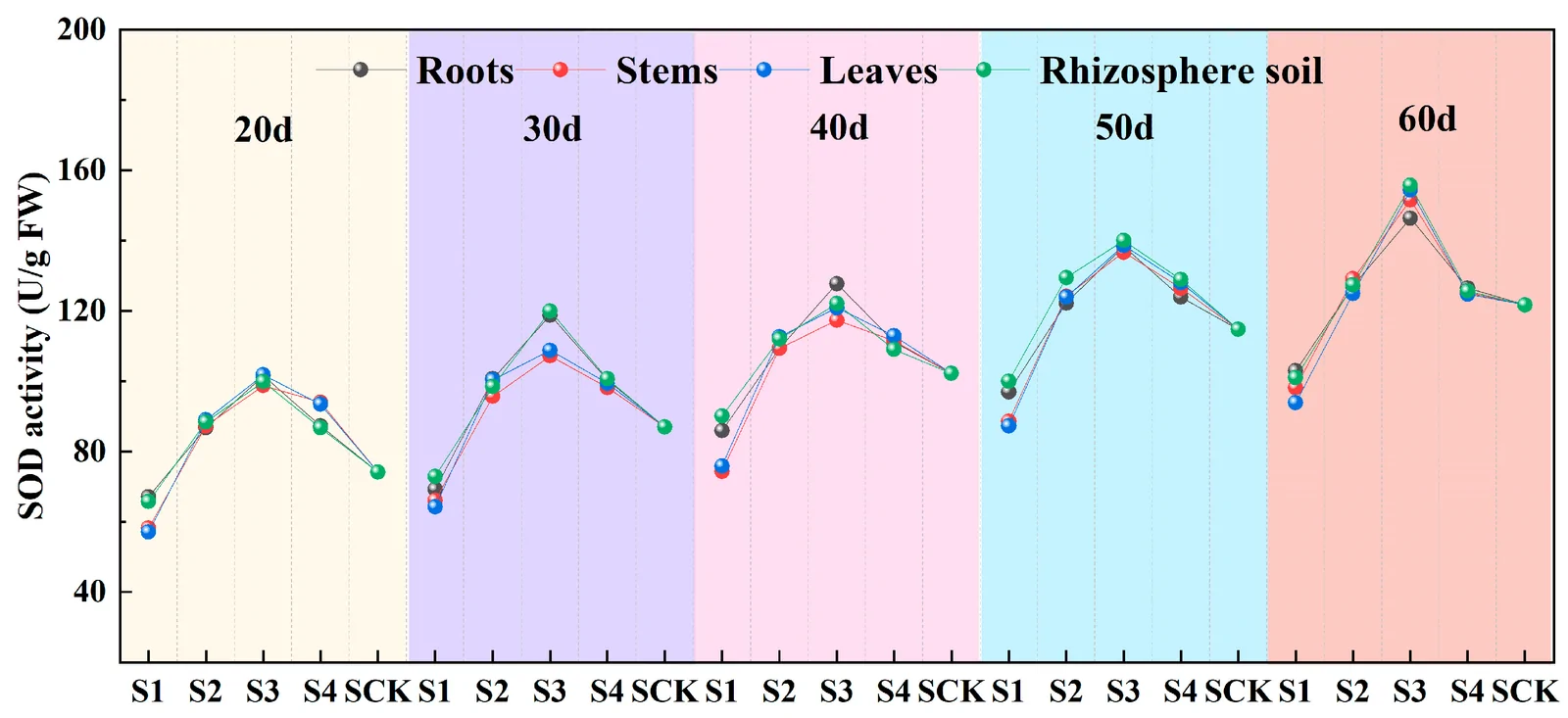
Effects of water extracts from different sorghum parts on SOD activity in giant foxtail.

The effects of water extracts from different sorghum parts on SOD activity in giant foxtail showed that the undiluted extracts significantly inhibited SOD activity, while the diluted extracts promoted it. Over time, SOD activity gradually increased, and the increases became stable as time went on. For root extracts, the S1 treatment showed no significant difference at 20 d, but significantly decreased SOD activity by 20.43%, 15.95%, 15.50%, and 15.45% from 30 d to 60 d, respectively. For stem extracts, the S1 treatment significantly inhibited SOD activity at all time points, with reductions ranging from 19.36% to 27.25%. For leaf extracts, the S1 treatment also showed continuous significant inhibition, with an inhibition rate of 26.17% at 30 d. For rhizosphere soil extracts, the S1 treatment significantly inhibited SOD activity at all time points except 20 d (Figure 4).

**Figure 5:**
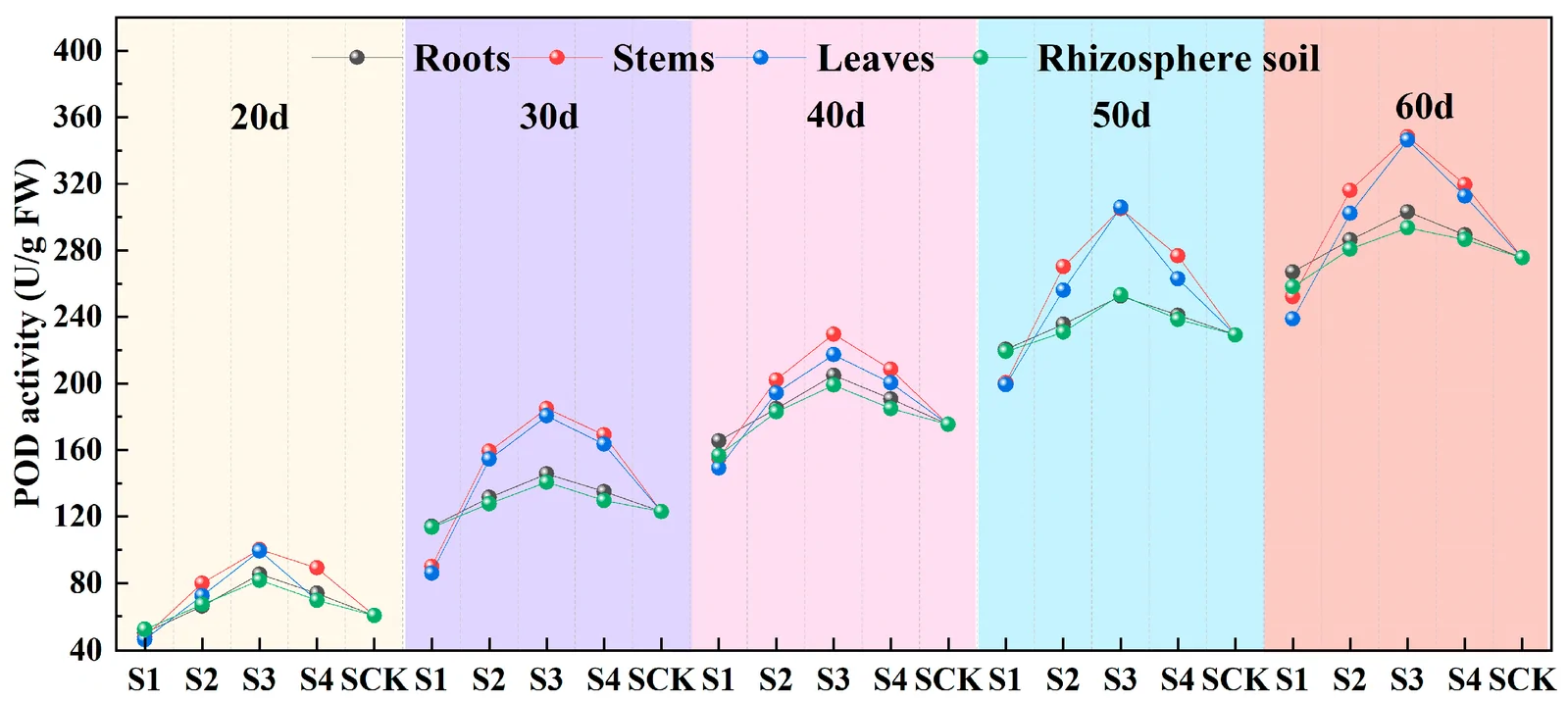
Effects of water extracts from different sorghum parts on POD activity in giant foxtail.

The effects of water extracts from different sorghum parts on POD activity in giant foxtail showed that the S1 treatments inhibited POD activity, while the diluted extracts promoted it. For root extracts, the S1 treatment was lower than the control at all time points, with a decrease of 16.89% at 20 d. For stem extracts, the S1 treatment showed stronger inhibition, with decreases ranging from 8.54% to 26.77% across all time points. For leaf extracts, the S1 treatment showed the most prominent inhibition, with decreases ranging from 13.10% to 30.11%. For rhizosphere soil extracts, the S1 treatment decreased POD activity by 13.41% at 20 d. All diluted extracts from different parts were significantly higher than the SCK, and the S3 treatment showed the strongest promoting effect (Figure 5).

**Figure 6:**
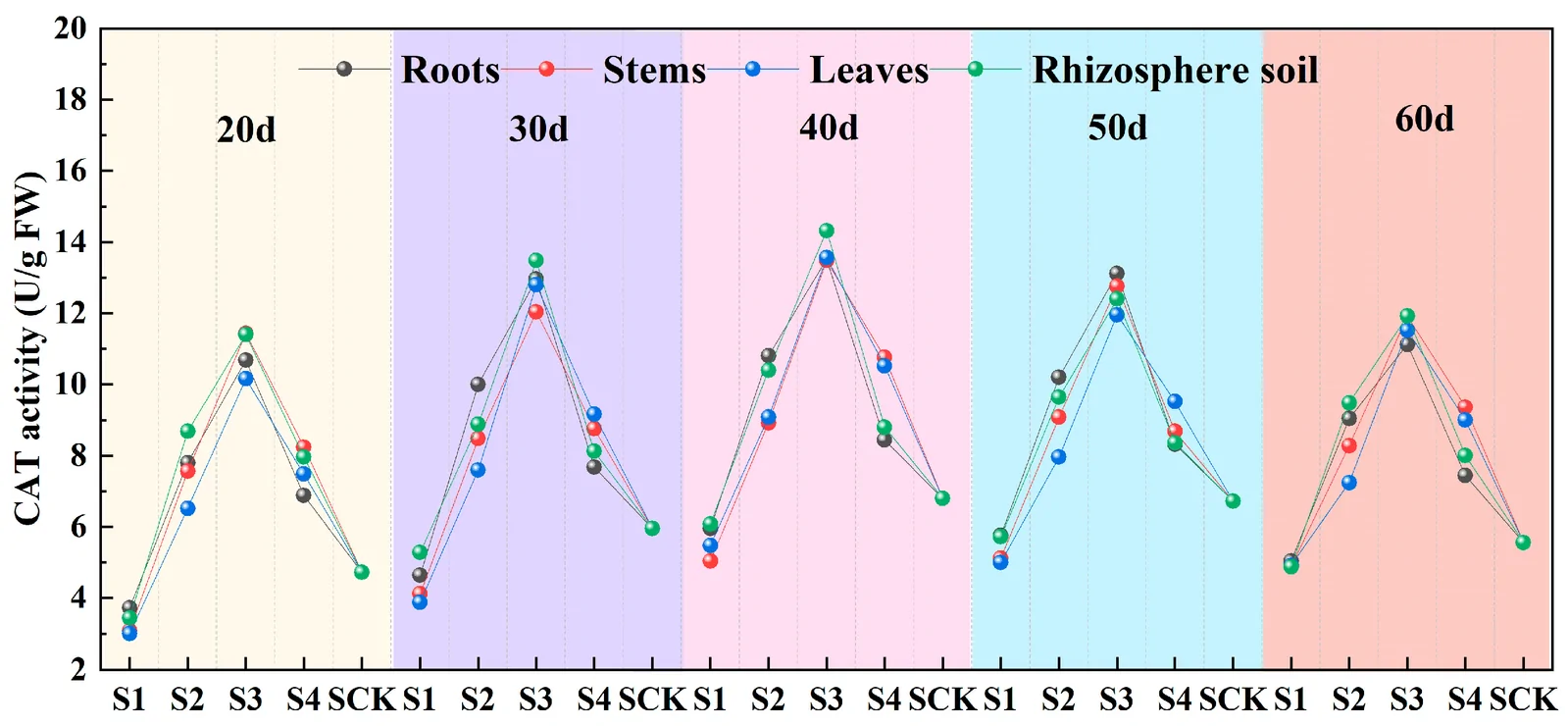
Effects of water extracts from different sorghum parts on CAT activity in giant foxtail.

The effects of water extracts from different sorghum parts on CAT activity in giant foxtail were significant. Under the S3 treatment, root extract increased CAT activity by 98.82% at 40 d, stem extract increased it by 89.88% at 50 d, leaf extract increased it by 117.96% at 40 d, and rhizosphere soil extract increased it by 114.39% at 60 d compared with the SCK. Leaf and rhizosphere soil extracts showed stronger effects than root and stem extracts. The S1 treatments generally showed inhibitory effects, with the strongest inhibition observed in stem and leaf extracts, while no clear effect was found on rhizosphere soil extracts. After dilution, the promoting effects increased, showing a clear concentration-dependent pattern (Figure 6).

**Figure 7:**
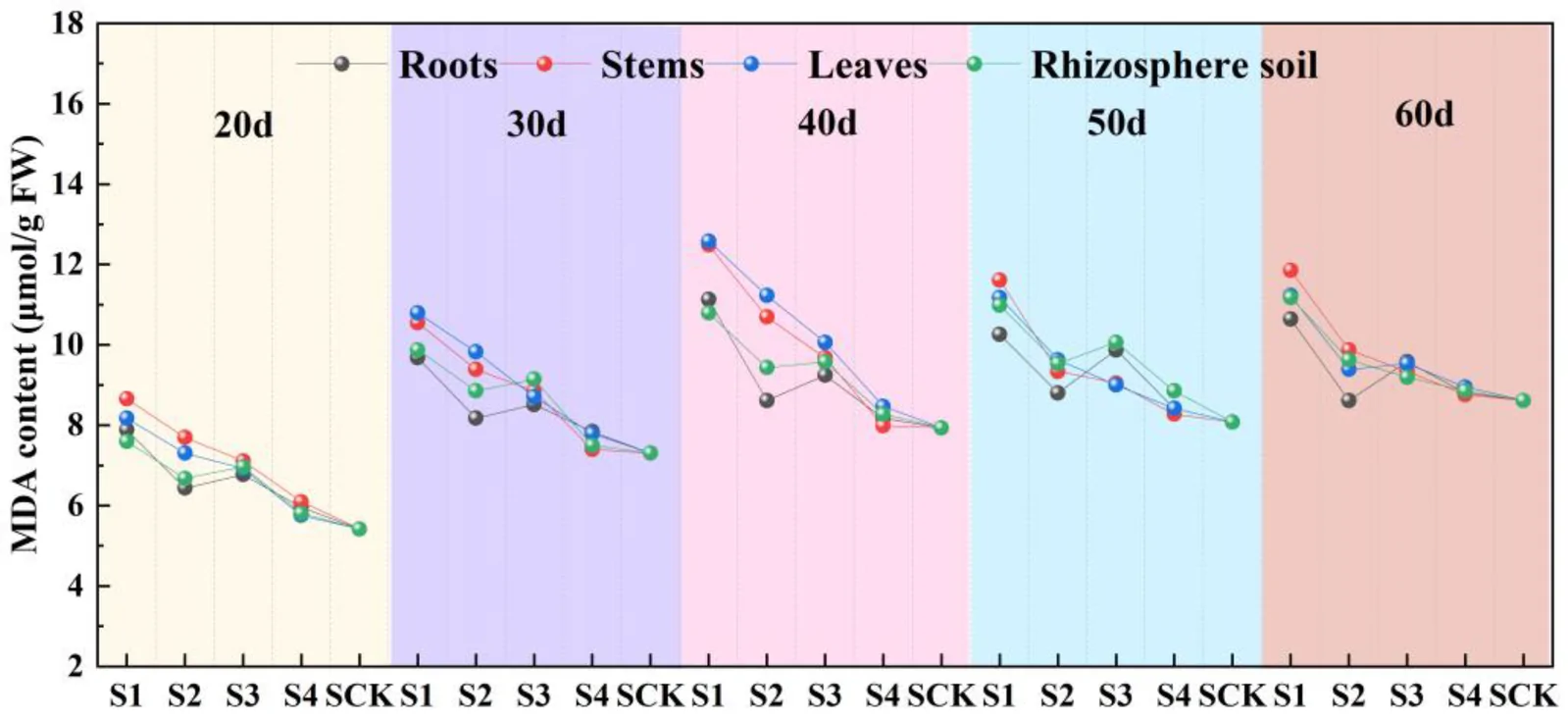
Effects of water extracts from different sorghum parts on MDA content in giant foxtail.

Under sorghum extract treatments, the MDA content in giant foxtail was higher than that in the SCK. As the dilution factor increased, MDA content decreased. Over time, the increases in MDA content first rose and then fell. For the S1 treatments, all parts showed significant increases. Root and stem extracts increased MDA content by 45.57% and 59.78% at 20 d, respectively. Leaf and rhizosphere soil extracts increased it by 58.62% and 36.02% at 40 d, respectively. The S2 and S3 treatments also promoted MDA content increases, but the effects were weaker than those of S1. Under the S4 treatments, MDA content generally showed no significant differences from the SCK (Figure 7).

## 3. Discussion

Seed germination marks the start of the plant life cycle and is the most sensitive stage for plants to respond to external chemical signals [22]. Germination rate is the most basic indicator for measuring seed germination ability. Germination energy reflects the speed and uniformity of germination, and the germination index provides a more complete measure of seed vigor [23]. In this study, extracts from different sorghum parts all showed low-concentration promotion and high-concentration inhibition, which is consistent with the dose–response relationship of allelopathy previously summarized by other researchers [24]. Some studies have found that water-soluble compounds released from crop roots can regulate the germination of nearby seeds, possibly by adjusting the balance of reactive oxygen species and signaling pathways inside the cells. For example, Gfeller et al. [25] reported that root exudates from buckwheat (Fagopyrum esculentum) suppressed the germination of redroot pigweed (Amaranthus retroflexus) through such mechanisms.

Seed germination and root and shoot growth are regulated by both internal and external factors. This stage is the most sensitive and is easily affected by environmental interference. Jie et al. [26] reported that seed germination and seedling growth in maize are regulated by complex physiological and transcriptional mechanisms in response to environmental signals. Inhibition of radicle growth can reduce root size and weaken the ability to absorb water and nutrients. Inhibition of plumule growth can lead to small and weak plants, causing them to lose their competitive advantage for light and ultimately limiting biomass accumulation. Similar effects have been observed in maize, where drought stress significantly inhibited radicle and plumule growth at the germination and seedling stages [27]. High-concentration undiluted treatments inhibited root and shoot growth. In particular, the sorghum root undiluted extract showed an inhibition rate of over 73% on root length, suggesting that it may contain components that inhibit cell wall synthesis or hormone signaling, thus blocking cell expansion [28]. In addition, the inhibition of shoot fresh weight by sorghum extracts was relatively weak, suggesting that the inhibitory effect was more related to cell elongation than to the accumulation of cellular materials [29].

Plant growth is closely related to environmental conditions. When the external environment changes, plants adjust their morphological structures to adapt to environmental stimuli. Lee et al. [30] reported that plants, including Arabidopsis (*Arabidopsis thaliana*), exhibit thermomorphogenic adaptation in response to elevated temperatures. Agronomic traits are the most direct indicators for evaluating plant growth, and in wheat (*Triticum aestivum* L.), traits such as plant height and biomass allocation are commonly used to assess drought tolerance [31]. As important external environmental factors, allelochemicals can induce a series of morphological and structural changes in plants, including reduced growth rate, altered biomass allocation, accelerated tissue senescence, and even plant death [32]. Studies have shown that exogenous phenolic acids (salicylic acid, ferulic acid, p-coumaric acid, and p-hydroxybenzoic acid) can significantly induce the allelopathic inhibitory effect of wild rice (*Oryza longistaminata*) accession S37 on barnyard grass (*Echinochloa crus-galli*). Specifically, treatment with 100 mg/L ferulic acid increased the inhibition rates of barnyard grass plant height and fresh weight by 38.12 and 26.31 percentage points, respectively, compared with the control. These results are consistent with our findings on the inhibitory effects of sorghum water extracts on giant foxtail growth, suggesting that phenolic allelochemicals can mediate concentration-dependent weed suppression across different crop–weed systems. This provides an important reference for the application of allelochemicals in ecological weed management [33]. Previous studies have found that different concentrations of Flaveria bidentis (*Flaveria bidentis* (L.) Kuntze) extracts significantly inhibited the plant height, root length, fresh weight, and dry weight of maize seedlings [34]. In this experiment, the S3 treatment generally showed the best promoting effects on plant height, stem diameter, and aboveground fresh and dry weight, while the undiluted treatments mostly significantly inhibited the aboveground growth and development of giant foxtail. Root, stem, leaf, and rhizosphere soil extracts all had allelopathic activity, but the promoting effects of stem and rhizosphere soil extracts were generally stronger. Over time, the promoting effects of all treatments on the agronomic traits of giant foxtail first increased and then became stable or slightly decreased.

Sorghum is known to produce a range of allelochemicals, among which sorgoleone is the most studied compound exuded from roots. Sorgoleone is a substituted p-benzoquinone [35]. In addition to sorgoleone, sorghum tissues contain various phenolic acids, including ferulic acid, p-hydroxybenzoic acid, vanillic acid, and caffeic acid [36], which are water-soluble and may contribute to the allelopathic effects observed in our water extract treatments. Unlike sorgoleone, which is primarily root-specific, these phenolic compounds are distributed in multiple plant parts, which is consistent with our finding that stem and leaf extracts also exhibited allelopathic activity.

SOD, POD, and CAT are the main antioxidant defense enzymes in plants. When plants are under stress, these enzymes can remove reactive oxygen species and reduce stress damage. The activities of these antioxidant enzymes can also reflect the level of stress on the plant. Stress conditions can induce membrane lipid peroxidation in plants, and the product MDA can damage proteins and nucleic acids by modifying their structures. Therefore, MDA content can be used to indicate the degree of cell membrane damage [37]. Some studies found that after treatment with cabbage leaf water extracts on zucchini, kidney bean, and maize, the activities of SOD, POD, and CAT increased at high concentrations, and the increase in MDA content indicated oxidative stress [38]. The results of this experiment are similar to previous findings. The S3 treatment significantly promoted the activities of SOD, POD, and CAT, suggesting that it activated the antioxidant system of giant foxtail, enhanced its ability to remove reactive oxygen species, and thus reduced oxidative stress [39]. The increase in MDA content reflected more severe membrane lipid peroxidation damage. Although MDA content in giant foxtail increased under sorghum extract treatments, it was accompanied by a significant rise in antioxidant enzyme activities, showing a balancing mechanism. Similar antioxidant responses have been observed in other plant–allelochemical systems. For instance, phenolic acid treatments in Trichosanthes kirilowii (*Trichosanthes kirilowii* Maxim.) induced significant increases in SOD, POD, and CAT activities, along with elevated MDA content, indicating that the activation of antioxidant enzymes is a common physiological response to allelochemical stress [40]. Likewise, allelopathic extracts from Wedelia species have been shown to increase antioxidant enzyme activities in receptor plants while simultaneously causing membrane lipid peroxidation, as reflected by increased MDA levels [41]. These examples, together with our findings, suggest that the upregulation of antioxidant enzymes represents a general defensive strategy in plants exposed to allelochemical stress, rather than a species-specific response.

In summary, this study systematically characterized the allelopathic effects of water extracts from different sorghum parts on giant foxtail and confirmed that the dilution level is the key factor determining the direction of the allelopathic response. Among the different parts, root extracts showed relatively strong activity; however, this conclusion is based solely on biological responses and lacks chemical identification and quantification of the specific active compounds in the extracts. In addition, the effects of rhizosphere soil extracts may be influenced by soil physicochemical properties and microbial metabolites, and thus cannot be attributed solely to sorghum allelopathy. Therefore, the conclusions of this study are mainly applicable to indoor conditions with water extract treatments. Future studies should combine chemical profiling techniques such as LC-MS or HPLC to identify the active components, further explore the underlying mechanisms at physiological and metabolic levels, and verify the practical effects under field conditions. Appropriate positive controls should also be included in future investigations to better quantify the allelopathic potential of sorghum extracts.

## 4. Materials and Methods

### 4.1. Experimental Materials

Tested crop: Sorghum (Jinza 22, bred by the Sorghum Research Institute of Shanxi Agricultural University).

Receptor plants: Giant foxtail seeds were collected from foxtail millet fields at the Agricultural Experiment Station of Shanxi Agricultural University.

The field experiment was carried out in 2025 at the experimental field of the Agricultural Experiment Station of Shanxi Agricultural University. Sorghum was sown in mid-May. After rotary tillage, the field was divided into plots with three replications, and conventional field management practices were carried out. Fresh crop plants at the seedling stage were collected in mid-to-late June for the preparation of water extracts (Table 5).

### 4.2. Preparation of Water Extracts

Healthy whole sorghum plants at the seedling stage were collected from sorghum fields. After removing impurities and shaking off dust, the plants were separated into roots, stems, and leaves. Each part was placed in a cool and dry place for natural air drying. The dried materials were cut into small pieces, ground with a grinder, and passed through a 100-mesh sieve. The dry powder was put into bags and stored in a refrigerator at 4 °C. Rhizosphere soil was collected using the root-shaking method, sieved, bagged, and stored in a refrigerator at 4 °C. The dry powder was mixed with distilled water at a mass ratio of 1:10 in beakers. The beakers were sealed with plastic film and placed in an ultrasonic cleaner for ultrasonic extraction for 30 min. After standing for 10 min, the mixture was filtered through double-layer gauze. The filtrate was then centrifuged at 8000 r/min for 5 min. The resulting supernatant was used as the stock solution (S1). The stock solution was diluted to 10-fold (S2), 50-fold (S3), and 100-fold (S4) dilutions, giving four concentration levels. All solutions were stored in a refrigerator at 4 °C until use. Each dilution was prepared immediately before use to avoid degradation of active components due to long-term storage.

### 4.3. Petri Dish Germination Test

Petri dishes with a diameter of 9 cm were used. Two layers of filter paper were placed in each dish and sterilized in an autoclave. Giant foxtail seeds with plump and uniform appearance were selected, soaked in 1% sodium hypochlorite for 10 min for surface sterilization, rinsed five times with sterile water, and then dried with sterile filter paper before use. Each dish received 5 mL of water extracts from different sorghum parts in the stock solution (S1), 10-fold dilution (S2), 50-fold dilution (S3), and 100-fold dilution (S4). Sterile distilled water was used as the blank control (SCK). Thirty giant foxtail seeds were evenly placed in each dish. The dishes were kept in a constant-temperature light incubator at 25 °C and 50% humidity. The corresponding extracts were added every 24 h according to the moisture condition of the dishes. The number of germinated seeds was recorded daily. Germination energy was counted on the 3rd day, and germination rate was counted on the 8th day. Root and shoot length and fresh weight of giant foxtail seedlings were measured. The allelopathic response index (RI) and synthetic allelopathic effect index (SE) were calculated for each indicator.

### 4.4. Pot Experiment

The pot experiment was conducted in the Laboratory of Crop Chemistry and Regulation at the College of Agriculture, Shanxi Agricultural University. Giant foxtail seeds with full grains and uniform size were selected, and 30 seeds were sown in each nutrient pot with dimensions of 5 cm × 5 cm × 8 cm. Every three pots were placed as one treatment in a seedling tray with dimensions of 25 cm × 10 cm × 10 cm, and then transferred to a greenhouse at 25 °C for cultivation. During the cultivation period, the light–dark cycle was set to 16 h of light and 8 h of darkness. Each treatment pot was irrigated with 400 mL of the corresponding extract every other day using a bottom watering method, and the control group was irrigated with water. When the seedlings had grown for about 15 days, thinning and soil covering were carried out, and finally, eight healthy and vigorous seedlings were kept in each pot. Plant samples of giant foxtail were collected and related indicators were measured at 20 d, 30 d, 40 d, 50 d, and 60 d after sowing.

### 4.5. Measurement Indicators and Methods

#### 4.5.1. Measurement and Calculation Methods for Seed Germination-Related Indicators

The number of germinated seeds in each dish was recorded at the same time every day. After 8 days of cultivation, the surface moisture of the seeds was removed with filter paper. Ten uniformly germinated seedlings were randomly selected from each dish. The roots and shoots of the germinated seeds were cut off with scissors. Root length and shoot length were measured with a ruler, and root fresh weight and shoot fresh weight were weighed using an analytical balance with an accuracy of 0.0001 g. The germination index (GI) of giant foxtail seeds was calculated based on the daily germination counts. Germination energy (GE) was calculated from the germination counts on the 3rd day, and germination rate (GR) was calculated from the germination counts on the 8th day. The corresponding allelopathic response index (RI) was also calculated.

Germination index (GI) = ∑(Gt/Dt)(1)

Germination energy (GE) = (G3/N) × 100%(2)

Germination rate (GR) = (G8/N) × 100%(3)

Allelopathic response index (RI) = 1 − CK/T (T ≥ C)(4)

Allelopathic response index (RI) = T/CK − 1 (T < C)(5)

Gt is the number of germinated seeds on a given day, Dt is the corresponding day, N is the total number of giant foxtail seeds in each dish, T is the treatment value, and CK is the control value.

The synthetic allelopathic effect index (SE) was calculated as the average of the allelopathic response indices (RI) for germination rate, root length, shoot length, root fresh weight, and shoot fresh weight of giant foxtail under each concentration of water extracts from roots, stems, leaves, and rhizosphere soil.

#### 4.5.2. Measurement and Calculation Methods for Indicators in the Pot Experiment

Giant foxtail plants with uniform growth were selected from each pot, with three plants per pot used for agronomic trait measurements. Plant height was measured with a tape measure from the base of the plant to the top of the flag leaf. Stem diameter was measured with a vernier caliper. The length and width of the second leaf from the top were measured with a ruler, and leaf area was calculated as leaf length × leaf width × 0.75. The aboveground fresh weight was weighed using an electronic analytical balance. The samples were placed in an oven and killed at 105 °C for 30 min, then dried at 80 °C to constant weight. The aboveground dry weight was then weighed.

Fresh samples of 0.1 g from the second leaf from the top of giant foxtail plants were collected at each time point and under each treatment. The samples were cut into pieces and ground on ice, then mixed with 1.8 mL of 0.05 mol L^−1^ phosphate buffer (pH 7.8). The homogenate was centrifuged at 12,000 rpm for 15 min at 4 °C. The supernatant was used for measuring the activities of SOD, POD, and CAT.

Superoxide dismutase (SOD) activity was measured using the nitroblue tetrazolium (NBT) photoreduction method, and the enzyme activity was calculated based on the percentage inhibition of absorbance at 560 nm [42]. Peroxidase (POD) activity was measured using the guaiacol method, and enzyme activity was determined by monitoring the increase rate of absorbance at 470 nm [43]. Catalase (CAT) activity was measured using the ultraviolet spectrophotometry method, and enzyme activity was determined by recording the decrease rate of absorbance at 240 nm [44]. Malondialdehyde (MDA) content was measured using the thiobarbituric acid method [45].

### 4.6. Data Processing and Analysis

Data were organized and analyzed using Microsoft Excel 2022 (Microsoft, Redmond, WA, USA) and IBM SPSS Statistics 25 (SPSS Inc., Chicago, IL, USA). Graphing and table preparation were performed using Origin 2024 (OriginLab, Northampton, MA, USA) and Microsoft Excel 2021. In the statistical analysis, one-way analysis of variance (ANOVA) was performed on data that met the homogeneity of variance assumption, and Duncan’s multiple range test was used for comparison among groups. Significance was set at *p* < 0.05.

## 5. Conclusions

This experiment studied the effects of water extracts from sorghum roots, stems, leaves, and rhizosphere soil on seed germination, seedling growth, agronomic traits, and physiological indicators of giant foxtail. The results showed that extracts from different parts had low-concentration promotion and high-concentration inhibition effects on germination indicators. The S3 treatment showed the best promoting effect. The S2 and S4 treatments generally promoted growth, but their effects were weaker than that of S3. The S1 treatment showed significant inhibition. All diluted treatments significantly increased plant height, stem diameter, leaf area, and biomass accumulation of giant foxtail seedlings. They also enhanced the activities of antioxidant enzymes such as SOD, POD, and CAT, and decreased MDA content. These results indicated that the diluted extracts could activate the plant defense system without causing oxidative damage. Among the different sorghum parts, root extracts had higher allelopathic activity than stem and leaf extracts. Leaf extracts showed strong promoting activity after high dilution, while stem and rhizosphere soil extracts had relatively mild allelopathic activity. This study revealed the “low-concentration promotion and high-concentration inhibition” pattern of sorghum allelochemicals on giant foxtail and the related antioxidant physiological mechanisms. It also identified roots as the main active part and S3 as the optimal concentration for promotion. These findings provide basic data and theoretical support for the development and utilization of sorghum allelochemicals and for the green control of giant foxtail. These findings provide a theoretical basis for the targeted extraction of sorghum allelochemicals and the development of ecological control strategies for giant foxtail.

## Figures and Tables

**Table 1:** Effects of water extracts from different sorghum parts on root and shoot growth of giant foxtail.

Parts	Treatments	Root Length (cm)	Root Fresh Weight (mg)	Shoot Length (cm)	Shoot Fresh Weight (mg)
Roots	S1	1.45 ± 0.14 c	0.57 ± 0.09 c	1.50 ± 0.07 c	3.13 ± 0.12 a
	S2	6.91 ± 0.39 a	1.42 ± 0.11 a	2.24 ± 0.18 a	3.23 ± 0.12 a
	S3	6.79 ± 0.45 a	1.29 ± 0.12 a	1.78 ± 0.16 b	2.01 ± 0.09 c
	S4	6.31 ± 0.34 a	1.25 ± 0.19 ab	2.43 ± 0.09 a	2.65 ± 0.20 b
	SCK	5.45 ± 0.35 b	1.02 ± 0.09 b	1.50 ± 0.06 c	2.15 ± 0.13 c
Stems	S1	—	—	—	—
	S2	4.56 ± 0.76 b	1.08 ± 0.28 b	1.45 ± 0.06 b	3.02 ± 0.20 a
	S3	5.09 ± 0.34 b	1.20 ± 0.13 b	1.53 ± 0.27 b	2.10 ± 0.16 b
	S4	6.87 ± 0.27 a	1.71 ± 0.17 a	2.50 ± 0.14 a	3.10 ± 0.14 a
	SCK	5.45 ± 0.35 b	1.02 ± 0.09 b	1.50 ± 0.06 b	2.15 ± 0.13 b
Leaves	S1	—	—	—	—
	S2	3.67 ± 0.57 d	1.10 ± 0.21 b	1.60 ± 0.16 b	2.73 ± 0.18 c
	S3	7.70 ± 0.46 b	2.26 ± 0.22 a	2.35 ± 0.05 a	3.49 ± 0.09 a
	S4	8.62 ± 0.24 a	2.07 ± 0.08 a	2.37 ± 0.02 a	3.20 ± 0.12 b
	SCK	5.45 ± 0.35 c	1.02 ± 0.09 b	1.50 ± 0.06 b	2.15 ± 0.13 d
Rhizosphere soil	S1	5.39 ± 0.27 c	1.83 ± 0.08 b	2.86 ± 0.15 a	3.52 ± 0.08 a
	S2	8.00 ± 0.54 a	2.57 ± 0.09 a	1.75 ± 0.30 b	2.62 ± 0.37 b
	S3	5.98 ± 0.20 c	1.54 ± 0.09 c	1.22 ± 0.02 c	1.75 ± 0.05 d
	S4	6.85 ± 0.34 b	1.91 ± 0.16 b	1.67 ± 0.20 b	2.47 ± 0.10 bc
	SCK	5.45 ± 0.35 c	1.02 ± 0.09 d	1.50 ± 0.06 bc	2.15 ± 0.13 c

Note: “—” indicates that no giant foxtail seeds germinated under the S1 treatments of stem and leaf extracts during the germination test, resulting in missing data for these treatments. Comparisons among different dilutions within the same plant part are shown, with lowercase letters indicating significant differences (*p* < 0.05).

**Table 2:** Effects of water extracts from different sorghum parts on the allelopathic index of giant foxtail seeds.

Parts	Treatments	Allelopathic Index (RI)					Synthetic Effects (SE)
		Germination Rate	Root Length	Root Fresh Weight	Shoot Length	Shoot Fresh Weight	
Roots	S1	−0.450	−0.733	−0.438	0.000	0.313	−0.262
	S2	−0.100	0.212	0.282	0.330	0.335	0.212
	S3	0.111	0.197	0.211	0.157	−0.065	0.122
	S4	0.200	0.136	0.182	0.382	0.191	0.218
Stems	S1	—	—	—	—	—	—
	S2	−0.150	−0.163	0.056	−0.031	0.289	0.000
	S3	0.075	−0.066	0.150	0.017	−0.022	0.031
	S4	−0.125	0.207	0.402	0.400	0.308	0.238
Leaves	S1	—	—	—	—	—	—
	S2	−0.275	−0.326	0.073	0.063	0.215	−0.050
	S3	−0.025	0.293	0.549	0.363	0.384	0.313
	S4	−0.125	0.368	0.508	0.368	0.329	0.290
Rhizosphere soil	S1	−0.025	−0.010	0.444	0.476	0.390	0.255
	S2	0.000	0.319	0.604	0.144	0.181	0.250
	S3	0.286	0.089	0.338	−0.187	−0.183	0.069
	S4	0.167	0.205	0.465	0.100	0.132	0.214

Note: “—” indicates that no giant foxtail seeds germinated under the S1 treatments of stem and leaf extracts during the germination test, resulting in missing data for these treatments. Comparisons among different dilutions within the same plant part are shown.

**Table 3:** Effects of water extracts from different sorghum parts on the aboveground fresh weight of giant foxtail (mg).

Parts	Treatment	Time				
		20 d	30 d	40 d	50 d	60 d
Roots	S1	5.54 ± 0.54 b	15.10 ± 0.37 b	21.23 ± 0.63 b	27.27 ± 0.68 c	33.12 ± 1.64 c
	S2	6.12 ± 0.47 ab	16.01 ± 0.83 b	21.75 ± 0.81 b	30.49 ± 0.62 b	36.60 ± 0.55 b
	S3	7.75 ± 0.38 a	17.72 ± 0.50 a	26.37 ± 0.63 a	34.34 ± 1.22 a	40.34 ± 0.26 a
	S4	5.57 ± 0.67 b	13.09 ± 0.36 c	20.86 ± 0.55 b	29.21 ± 0.98 bc	32.78 ± 1.25 c
	SCK	4.46 ± 0.75 b	10.03 ± 0.42 d	17.61 ± 0.37 c	22.78 ± 1.10 d	26.05 ± 0.61 d
Stems	S1	5.45 ± 0.71 bc	11.11 ± 0.57 b	21.36 ± 0.41 c	33.23 ± 1.10 b	36.89 ± 0.89 b
	S2	7.29 ± 0.36 b	11.41 ± 0.65 b	24.89 ± 0.59 b	35.26 ± 0.93 ab	40.69 ± 0.44 a
	S3	9.75 ± 0.21 a	17.06 ± 0.59 a	29.56 ± 0.4 a	36.38 ± 0.39 a	41.86 ± 1.29 a
	S4	6.89 ± 0.71 b	11.16 ± 0.43 b	26.16 ± 0.48 b	29.21 ± 0.79 c	37.15 ± 1.06 b
	SCK	4.46 ± 0.75 c	10.03 ± 0.42 b	17.61 ± 0.37 d	22.78 ± 1.10 d	26.05 ± 0.61 c
Leaves	S1	4.96 ± 0.18 bc	17.45 ± 0.94 a	27.01 ± 1.02 b	33.6 ± 0.81 b	38.19 ± 0.96 c
	S2	6.60 ± 0.59 ab	15.10 ± 0.6 ab	25.75 ± 0.54 b	39.99 ± 0.96 a	44.22 ± 0.50 b
	S3	8.28 ± 0.58 a	13.08 ± 0.87 bc	34.08 ± 0.85 a	42.97 ± 1.41 a	47.90 ± 0.80 a
	S4	5.87 ± 0.47 bc	11.74 ± 0.85 cd	26.44 ± 1.02 b	40.56 ± 1.00 a	42.40 ± 0.60 b
	SCK	4.46 ± 0.75 d	10.03 ± 0.42 d	17.61 ± 0.37 c	22.78 ± 1.10 c	26.05 ± 0.61 d
Rhizosphere soil	S1	5.65 ± 0.44 b	13.10 ± 0.41 ab	23.71 ± 1.36 b	33.45 ± 1.13 b	40.71 ± 0.39 ab
	S2	5.25 ± 0.35 b	12.85 ± 0.49 ab	23.6 ± 0.63 b	34.67 ± 0.74 ab	38.15 ± 1.24 b
	S3	7.97 ± 0.37 a	14.46 ± 0.55 a	31.18 ± 0.88 a	36.45 ± 0.61 a	40.98 ± 0.57 a
	S4	5.31 ± 0.47 b	11.58 ± 0.71 bc	21.84 ± 0.87 b	28.67 ± 0.68 c	35.19 ± 1.07 c
	SCK	4.46 ± 0.75 b	10.03 ± 0.42 c	17.61 ± 0.37 c	22.78 ± 1.10 d	26.05 ± 0.61 d

Note: Comparisons among different dilutions within the same plant part on the same day are shown, with lowercase letters indicating significant differences (*p* < 0.05).

**Table 4:** Effects of water extracts from different sorghum parts on the aboveground dry weight of giant foxtail (mg).

Parts	Treatment	Time				
		20 d	30 d	40 d	50 d	60 d
Roots	S1	0.61 ± 0.05 b	3.36 ± 0.33 a	4.05 ± 0.09 b	6.33 ± 0.31 b	9.76 ± 0.44 b
	S2	0.70 ± 0.05 b	3.68 ± 0.33 a	4.68 ± 0.39 b	7.11 ± 0.14 b	10.37 ± 0.32 ab
	S3	0.89 ± 0.01 a	3.93 ± 0.16 a	6.07 ± 0.28 a	9.50 ± 0.42 a	11.56 ± 0.13 a
	S4	0.66 ± 0.05 b	2.18 ± 0.04 b	4.29 ± 0.28 b	6.44 ± 0.49 b	8.30 ± 0.61 c
	SCK	0.55 ± 0.08 b	1.83 ± 0.09 b	3.89 ± 0.15 b	5.02 ± 0.11 c	6.90 ± 0.40 d
Stems	S1	0.64 ± 0.07 b	1.92 ± 0.27 b	5.70 ± 0.19 b	9.18 ± 0.33 a	10.38 ± 0.48 ab
	S2	0.76 ± 0.06 ab	1.79 ± 0.19 b	5.63 ± 0.35 b	7.07 ± 0.23 b	10.95 ± 0.35 a
	S3	1.06 ± 0.07 a	3.20 ± 0.16 a	6.89 ± 0.24 a	8.69 ± 0.30 a	10.72 ± 0.90 a
	S4	0.75 ± 0.17 ab	2.03 ± 0.33 b	5.32 ± 0.48 b	6.91 ± 0.25 b	8.51 ± 0.78 bc
	SCK	0.55 ± 0.08 b	1.83 ± 0.09 b	3.89 ± 0.15 c	5.02 ± 0.11 c	6.90 ± 0.40 c
Leaves	S1	0.61 ± 0.01 b	2.72 ± 0.42 ab	5.97 ± 0.54 ab	9.50 ± 0.48 b	12.52 ± 0.44 b
	S2	0.78 ± 0.09 b	2.33 ± 0.32 ab	5.22 ± 0.53 bc	11.45 ± 0.56 ab	11.5 ± 0.99 b
	S3	1.25 ± 0.04 a	3.19 ± 0.39 a	7.48 ± 0.76 a	12.07 ± 1.00 a	14.92 ± 0.23 a
	S4	0.74 ± 0.10 b	3.00 ± 0.46 ab	5.84 ± 0.35 ab	10.01 ± 0.65 ab	11.71 ± 0.38 b
	SCK	0.55 ± 0.08 b	1.83 ± 0.09 b	3.89 ± 0.15 c	5.02 ± 0.11 c	6.90 ± 0.40 c
Rhizosphere soil	S1	0.58 ± 0.05 b	3.80 ± 0.33 a	5.00 ± 0.34 ab	7.22 ± 0.64 ab	9.44 ± 0.67 a
	S2	0.58 ± 0.06 b	2.78 ± 0.14 b	4.88 ± 0.55 ab	6.30 ± 0.34 bc	9.45 ± 0.74 a
	S3	1.27 ± 0.20 a	4.18 ± 0.42 a	6.18 ± 0.47 a	8.17 ± 0.52 a	10.24 ± 0.57 a
	S4	0.53 ± 0.06 b	2.13 ± 0.37 b	4.01 ± 0.48 b	6.38 ± 0.36 bc	9.13 ± 0.47 a
	SCK	0.55 ± 0.08 b	1.83 ± 0.09 b	3.89 ± 0.15 b	5.02 ± 0.11 c	6.90 ± 0.40 b

Note: Comparisons among different dilutions within the same plant part on the same day are shown, with lowercase letters indicating significant differences (*p* < 0.05).

**Table 5:** Soil nutrient contents in the 0–20 cm soil layer before sowing in 2025.

Year	Preceding Crop	pH	Total P (g/kg)	Total K (g/kg)	Total N (g/kg)	Available K (g/kg)	Hydrolyzable N (g/kg)	Available P (g/kg)	Organic Matter (g/kg)
2025	Foxtail millet	8.16	1.15	22.69	1.05	297.59	70.64	12.40	25.34

## Data Availability

The data presented in this study are part of an ongoing master’s thesis project and are therefore not publicly available at this stage. All key data supporting the conclusions of this manuscript are fully presented in the figures and tables. For now, the data can be requested from the corresponding author for scientific communication and validation purposes.
